# Fermentation of brewer’s spent grains by *Pleurotus ostreatus*: process optimization by response surface methodology

**DOI:** 10.1186/s40643-026-01018-3

**Published:** 2026-05-14

**Authors:** Victoria-Luisa Hrazdil, Paula Hallmann, Josephine Dresler, Marco A. Fraatz, Holger Zorn

**Affiliations:** 1 Institute of Food Chemistry and Food Biotechnology, Justus Liebig University Giessen, Heinrich-Buff-Ring 17, 35392 Giessen, Germany; 2 Fraunhofer Institute for Molecular Biology and Applied Ecology, Ohlebergsweg 12, 35392 Giessen, Germany

**Keywords:** Brewer’s spent grains, Submerged fermentation, *Pleurotus ostreatus*, Response surface methodology

## Abstract

To achieve high-value utilization of brewer’s spent grains and to produce protein-rich food ingredients, brewer’s spent grains were upcycled by a liquid fungal fermentation and the fermentation conditions were optimized. Brewer’s spent grains (BSG) represent the most abundant by-product of the brewing industry. Although BSG are food-grade, their direct use in food is limited because of sensory changes of the products. In this study, screening experiments revealed that black beer spent grains could be efficiently upcycled by submerged fermentation with the edible fungus *Pleurotus ostreatus*. The conditions of the fermentation of BSG with *P. ostreatus* were optimized using response surface methodology, including the parameters substrate concentration, inoculum volume, initial pH value, and temperature. As no separation between BSG and mycelium was possible after the fermentation, ergosterol was used as a biomarker to determine the fungal growth. As optimum conditions, a BSG concentration of 17 g L^− 1^ dry matter, an inoculum volume of 4.4% (v/v), an initial pH of 9.3 and a temperature of 30 °C were identified. The fermentation with *P. ostreatus* increased the true protein content of the biomass compared to the spent grains and reduced the total fat content. The biological value was increased from 88 to 94 (reference standard is whole chicken egg with a biological value of 100). Tryptophan and lysine were limiting in the non-fermented spent grains, while after fermentation, the chemical score of lysine increased from 78 to 92, and tryptophan wasn’t limiting anymore. Fermentation by *P. ostreatus* enhanced the nutritional value of BSG, and the fermentation conditions were optimized by using response surface methodology.

**Graphical abstract:**

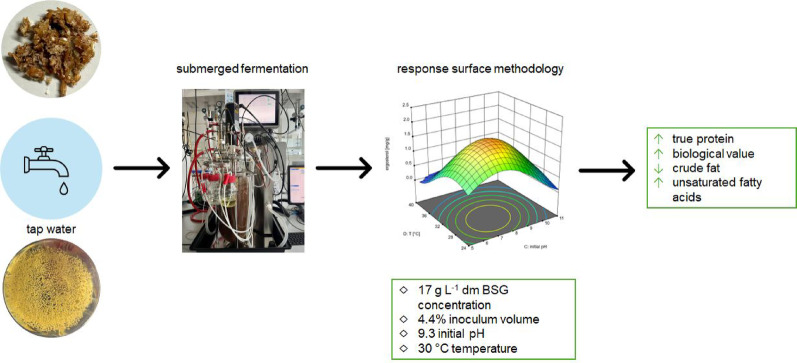

**Supplementary Information:**

The online version contains supplementary material available at 10.1186/s40643-026-01018-3.

## Introduction

Brewer’s spent grains (BSG), mostly consisting of barley, represent the dominating by-product of the brewing industry. During the beer production process, malt is crushed, mixed with water and heated in a controlled manner. In a filtration step known as lautering, the liquid wort is separated from the extracted malt, the spent grains (Ortiz et al. 2019). Compared to the other by-products, BSG account for the largest share of ~ 85%. For every 100 L of beer, about 20 kg of BSG accrue (Mussatto et al. 2006; Ortiz et al. 2019). The main components of BSG are husks, pericarp, and seeds. These contain mainly cellulose, hemicelluloses, and lignin, while the starch originally contained in the grain is hydrolyzed during the mashing process. Apart from the high crude fiber content, BSG contain ~ 22% protein, and around 30% of the protein consists of essential amino acids. The exact composition of the spent grains is influenced by the raw materials and the respective brewing process (Mussatto et al. 2006; Mussatto 2014).

Due to its high moisture content of about 80%, BSG is perishable and unsuitable for long storage. Currently, it is mostly used as feed and therefore sold to local farmers. Otherwise, it is used for energy production or as compost (Ortiz et al. 2019). In Europe, 70% of the produced BSG is used as feed, 10% is used for biogas production, and the rest is composted (Bianco et al. 2020). The use of BSG in food has been investigated in some studies. Spent grains were for example evaluated in baked goods like bread and cookies and as an ingredient in pasta or snack articles. Because of its color and possible changes to the products in terms of taste and texture, the usage of BSG as an ingredient in food is limited (Mussatto 2014; Belardi et al. 2025). Other applications of BSG included the usage in the production of bioplastic, in biofuel production, in hydrogen production or usage as adsorbent. Due to their properties, BSG represent a suitable substrate for microbial cultivation. In recent years, several processes have been described for the production of ethanol, lactic acid and xylitol, for example (Mussatto and Roberto 2005; Mussatto et al. 2007; Lisci et al. 2024; Belardi et al. 2025). The cultivation of edible fungi on BSG has also been studied (Wang et al. 2001).

More than 2000 species of mushrooms are known, some of which are used as edible or medicinal mushrooms. Mushrooms are well suitable for human nutrition as they contain a low energy content, high concentrations of crude fiber and bioactive substances such as antioxidants and *β*-glucan (Kalač 2013; Valverde et al. 2015). One of the most cultivated edible mushrooms worldwide is *Pleurotus ostreatus* (POS), a white rot fungus that can degrade lignin, hemicelluloses, and cellulose (Ritota and Manzi 2019). *P. ostreatus* has already been cultivated on several side streams, such as paper, various plant fibers, and sawdust. Fruiting bodies have also been cultivated on spent grain (Wang et al. 2001; Mandeel et al. 2005; Koutrotsios et al. 2014; Berger et al. 2022), and differences in the chemical composition of the fruiting bodies have been observed depending on the substrate used (Tshinyangu 1996).

An alternative to fruiting body production is the cultivation of mushrooms in liquid culture. The production of fruiting bodies is time-consuming and requires a relatively large amount of space, whereas liquid cultures generate more uniform mycelia in a shorter time. In general, liquid cultures are well suitable for enzyme production or waste conversion. The advantage compared to fruiting body cultivation is that the temperature, the pH value, and the aeration rate can be precisely monitored and regulated in liquid cultures (Abdullah et al. 2013; Corrêa et al. 2016).

Compared to yeasts or plant cells, mycelia typically contain protein with high biological values. By using side streams of the food industry as a source of nutrients and the possible reuse of processed fermentation liquids, processes can be designed in the sense of a circular economy (Berger et al. 2022).

One of the United Nation’s Sustainable Development Goals is to end hunger, food insecurity and malnutrition worldwide by 2030. In 2023, around 9% of the world’s population suffered from hunger. Around 29% of the global population was food insecure, and millions of people are still expected to be undernourished by 2030 (FAO et al. 2024). Reducing food losses and food waste is important to reach the goal of ending hunger, food insecurity and malnutrition. In 2019 about 14% of worldwide produced food was lost, but this does not include the food waste of the retail stage and consumers (FAO 2019). In line with the principles of a circular economy, by-products from the food industry should be reintroduced into the cycle and made usable for human consumption. Since BSG are produced in large quantities worldwide and their use in food has been very limited to date, research should be conducted to make them suitable for human consumption. The aim of the present study was thus to use BSG as a substrate for fungal liquid fermentation and to optimize the fermentation conditions by using response surface methodology.

## Materials and methods

### Microorganism and inoculum preparation

The fungus used in this study was *Pleurotus ostreatus* (Gö 508) and sourced from Georg-August Universität Göttingen (Germany). ITS sequencing confirmed its identification as *P. ostreatus*. POS was cultivated on malt extract agar (MEA, 20 g L^− 1^ malt extract, 15 g L^− 1^ agar agar) at 24 °C in darkness.

For the pre-cultures, a piece of mycelium (approx. 0.5 cm^2^) was excised and transferred into 100 mL of sterile malt extract medium (20 g L^− 1^) in a shaking flask. After homogenization by Ultra Turrax (IKA, Staufen im Breisgau, Germany 30 s, 10,000 rpm), the pre-culture was incubated on an orbital shaker (150 rpm, 25 mm shaking diameter) at 24 °C for 7 days in the dark. Prior to inoculation of the main cultures, the pre-culture was homogenized by Ultra Turrax.

### Culture medium and bioreactor

As main culture medium, brewer’s spent grains (BSG) of black beer and tap water were used. BSG were supplied by the Störtebeker Braumanufaktur (Germany) and shipped frozen. For medium preparation, the required amount of BSG and tap water were mixed in a Thermomix TM5 (Vorwerk, Wuppertal, Germany) at a speed of 5,800 rpm for 30 s. Afterwards, the medium was made up to 1 L, transferred into a bioreactor and autoclaved at 121 °C for 20 min. A parallel bioreactor system consisting of four vessels was used (Supplementary Table 1).

The medium was saturated with oxygen, and the desired pH-value was adjusted with 1 M NaOH. Subsequently, the DO-sensor was calibrated. During the fermentations, agitation and flow were regulated by a cascade (Supplementary Table 2) to hold the setpoint of 30% DO. The fermentations were stopped after four days of cultivation. The fermentates were harvested through a straining cloth and sieve. After harvesting, the biomass was freeze-dried, and the dry mass was estimated gravimetrically.

### Determination of ergosterol content

The dry biomass was analyzed for its ergosterol content according to Bickel Haase et al. with minor modifications (Bickel Haase et al. 2024). In this study, the dry biomass was milled by hand with mortar and pestle. Approximately 200 mg was weighed into a Pyrex tube. 50 mg of sodium ascorbate, 0.25 mL of internal standard, and 5 mL methanolic NaOH were added. Saponification, extraction, and silylation were performed according to Bickel Haase et al. (Bickel Haase et al. 2024). Samples were analyzed by GC-FID (7890 A, Agilent Technologies, Santa Clara, USA) with an injection volume of 1 µL and a split ratio of 5:1. A DB5ms column [(30 m x 0.32 mm x 0.25 μm, Agilent Technologies; temperature program: 100 °C (3 min)/ 30 °C min^− 1^/ 280 °C (12 min)/ 30 °C min^− 1^/ 320 °C (5 min)] with hydrogen as carrier gas (2 mL min^− 1^, constant flow) was used for separation.

### Experimental design

For optimization of the fermentation conditions by a response surface method, the concentration of BSG, the inoculum volume, the initial pH, and the temperature were chosen as factors. As responses, the ergosterol content of the biomass [mg g^− 1^] as well as the ergosterol content per liter fermentation broth [mg L^− 1^] were calculated. An overview over the factors and their ranges is shown in Supplementary Table 3. The aim of the optimization was to increase the ergosterol content and to simultaneously decrease the inoculum volume. An I-optimal design was chosen, and 52 runs in 3 blocks were performed, including eight replicates, nine lack of fit points, and five centre points. All runs and results are attached in Supplementary Table 4.

#### Treatment of data and confirmation

The results of all 52 runs were calculated and implemented into the experimental plan. All obtained responses were submitted to analysis of variance (ANOVA). The model was calculated by “Design Expert 13“ (Version 23.1.3, Stat-Ease, Minneapolis, USA). To confirm the model, four confirmation runs were performed under the optimized conditions. Run 21 wasn’t included in the calculations as the flow couldn’t be increased during the fermentation, and thus oxygen was not sufficiently transferred into the medium.

#### Quantification of fungal content

The fungal content of the fermentates was quantified via the ergosterol content. As a reference, *P. ostreatus* was cultivated under optimized conditions in 20 g L^− 1^ malt extract medium. The ergosterol content of the mycelium cultivated in malt extract medium referred to a fungal content of 100%.

#### Determination of crude protein

The determination of the crude protein content was performed according to Kjeldahl (Kjeldahl 1883). The oxidative acid digestion was carried out in a digestion block (Turbotherm, C. Gerhardt GmbH & Co. KG, Königswinter, DE). Steam distillation and titration were carried out automatically (Vapodest 450, C. Gerhardt GmbH & Co. KG, Supplementary Table 5). The calculation of the crude protein content was carried out by using 5.7 as Kjeldahl-factor for grains. Crude protein contents were evaluated in the screening experiments to compare the degradation of BSG by different fungi. As the dry biomass contains different types of BSG as well as different amounts of BSG, the crude protein contents are higher than the true protein contents. For the determination of the true protein content of the final product, an individually determined factor was used.

#### Determination of crude ash

The crude ash was determined after complete ashing of the sample at 550 °C by differential weighing.

#### Determination of crude fat

To determine the fat content, the samples were automatically digested with 4 M hydrochloric acid (Hydrotherm, C. Gerhardt GmbH & Co. KG). The fat was collected in a filter, and the filter was washed neutrally (Supplementary Table 6). After drying, the filters were automatically extracted with petroleum ether (boiling range 40–65 °C, Soxtherm, C. Gerhardt GmbH & Co. KG, Supplementary Table 7). The extracted fat was dried until mass constancy.

#### Determination of total carbohydrates

The total carbohydrate content was calculated considering the true protein, ash and fat contents.

#### Determination of amino acid composition

The analysis of the amino acids was carried out according to (Ahlborn et al. 2019). After sample preparation, the amino acids were separated on a cation exchange column (SYKAM GmbH, Eresing, Germany, Supplementary Table 8). For detection, the amino acids were post-column derivatized with ninhydrin to form colored compounds, which were detected with a UV/VIS detector at 570 nm and 440 nm (for proline), respectively. The gradients for the separation and the temperature programs are shown in Supplementary Fig. 1. The amino acids were quantified by an external five-point calibration.

The calculation of the amino acid amounts, biological values, and conversion factors for the true protein content was performed according to Ahlborn et al. (Ahlborn et al. 2019).

#### Determination of fatty acid composition

The fatty acid distribution was determined by derivatization to fatty acid methyl esters (FAME) according to Palmquist and Jenkins with minor modifications (Palmquist and Jenkins 2003). 0.5 g of freeze-dried and ground sample was weighed into a Pyrex tube and mixed with 2 mL nonadecanoic acid (2.0 mg mL^− 1^ in heptane) and 3 mL 10% methanolic hydrochloric acid. After thorough mixing using a vortex mixer, the sample was incubated for 2 h at 90 °C in a water bath and then cooled to room temperature. After the addition of 1 mL hexane and 10 mL 6% potassium carbonate solution, mixing was repeated. The sample was transferred to a headspace vial (20 mL) and centrifuged at 1500 *g* for 5 min. The organic phase was transferred to a fresh vial, sodium sulfate and activated charcoal were added and the sample was centrifuged again. The clear liquid was transferred to a brown glass vial (2 mL) and analyzed using an Agilent 7890B gas chromatograph (GC) with coupled Agilent 5977B mass spectrometer (MS). 1 µL was injected into a split-splitless (S/SL) inlet (250 °C) with a split rate of 100:1. For FAME separation, a polar Agilent VF-WAXms column (30 m x 0.25 mm x 0.25 μm) was used with a constant helium flow (1.2 mL min^− 1^) and temperature program (40 °C (3 min)/ 5 °C min^− 1^/ 240 °C (12 min). After the GC column, the carrier gas flow was analyzed in the MS (transferline temperature: 250 °C, electron ionization energy: 70 eV, ion source temperature: 230 °C, quadrupole temperature: 150 °C, scan range: *m/z* 33–440). A standard mixture was also analyzed by GC-MS as a reference.

## Results and discussion

### Fungal strain selection

**Fig. 1:**
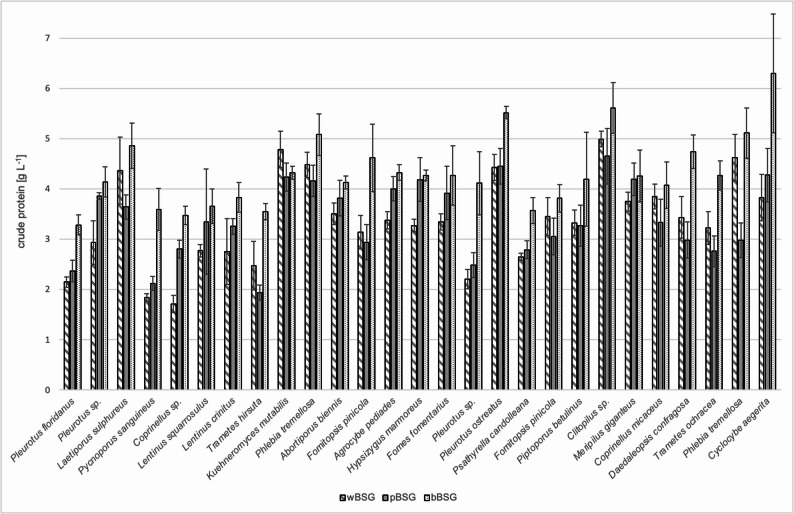
Crude protein contents of different fungi screened on wheat beer spent grains (wBSG), Pilsener beer spent grains (pBSG) and black beer spent grains (bBSG)

In a broad screening, 144 fungi from the institute’s culture collection were screened on agar plates with the different spent grains as sole nutrient source. 27 candidates were chosen for a subsequent screening in submerged culture due to their fast growth (> 70% coverage of the agar plate) after 7 days, a very dense mycelium or a meaty, savoury flavour (data not shown). A comparison of the crude protein contents of the different fungi cultivated on different spent grains showed that *P. ostreatus*, *Meripilus giganteus*, and *Cyclocybe aegerita* produced the highest values on black beer spent grains (Fig. 1). As *P. ostreatus* is a well-known edible fungus, its cultivation on black beer spent grains was further investigated.

### Optimization of the fermentation with DOE

For the ergosterol content of the biomass [mg g^− 1^], a quadratic model fitted best. By using „Design Expert 13“, all non-significant terms with a *p*-value > 0.05 were eliminated and therefore not included in the calculations. Elimination was performed backwards, beginning with the terms with the highest-level interactions. After calculations were done, Box-Cox-plot recommended a transformation by square root. The equation of the transformed model for the ergosterol content of the biomass [mg g^− 1^] is shown in Eq. (1). The terms A, B, C, and D are describing the concentration of BSG, the inoculum volume, the initial pH, and the temperature. AD, BD, and CD refer to the interactions between those parameters.

1 $$ \begin{aligned} \sqrt {{\mathrm{Ergosterol}}\left[ {{\mathrm{mg}}\:{\mathrm{g}}^{{ - 1}} } \right]} & = + 0.8951 - 0.3264\;{\mathrm{A}} \\ & \quad + 0.2520\:{\mathrm{B}} - 0.0687\:{\mathrm{C}} \\ & \quad - 0.5086\:{\mathrm{D}} - 0.1695\:{\mathrm{AD}} \\ & \quad + 0.1790\:{\mathrm{BD}} + 0.2542\:{\mathrm{CD}} \\ & \quad - 0.5189\:{\mathrm{C}}^{2} - 0.5261\:{\mathrm{D}}^{2} \\ \end{aligned} $$

The fit statistic for the model of the ergosterol content in the biomass [mg g^− 1^] is shown in Supplementary Table 9. The predicted R^2^ (0.2) was not as close to the adjusted R^2^ (0.5) as expected (expectation is < 0.2). This might be caused by a block effect. While carrying out the experiments, all factors that should not be evaluated, should be kept constant. Otherwise, their variations might influence the responses and thus influence the model. The fermentations in this study were carried out in four identical reactors over a period of several months and it was assumed that there was no difference between the reactors. It was also assumed that there were no changes in the other parameters over the period of the experiments. The fermentations and the downstream processing, including analysis, were always carried out in the same way. However, it cannot be excluded that minor changes did occur. Grouping the test into blocks by week or by bioreactor could reduce such potential influencing factors and reduce the distance of predicted R^2^ and adjusted R^2^. During the experiments, the factor space was expanded twice in terms of initial pH and temperature. Therefore, the data was already divided into three blocks. For the sake of clarity, the data was not grouped into further blocks The signal-to-noise-ratio was high with a value of 15 which is desirable for navigating the design space.

**Fig. 2:**
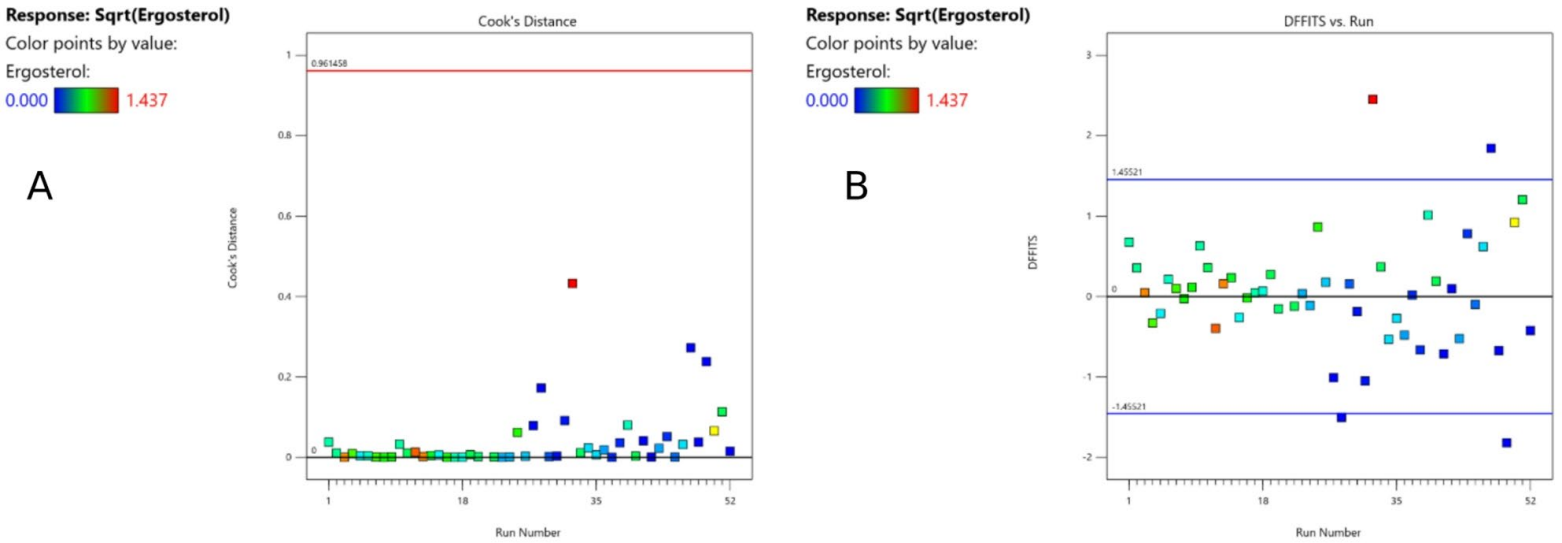
Cook’s distance (**A**) and difference of fits (DFFITS,** B**) for the ergosterol content of the biomass [mg g^-1^]

In the difference of fits test (DFFITS), four data points were outside the boundaries. However, these points were within the limit of Cook’s Distance and therefore used for the calculations (Fig. 2). By analyzing the figure with predicted versus actual values, no value was spotted as outlier (Fig. 4A). Therefore, all 52 runs (except for run 21) were used for calculating the model.

In the analysis of variance (ANOVA) in the supplementary material, all terms relevant for the calculation of the model are listed (Supplementary Table 10). The non-significant terms as pH and correlation between BSG concentration and temperature were used for hierarchical reasons. The model was significant and showed a non-significant lack of fit.

For the ergosterol content [mg L^− 1^], a cubic model was chosen due to its best fit. Like the ergosterol content of the biomass, all non-significant terms with* p*-values > 0.05 were eliminated, starting at the terms describing the interactions of the highest order. The Box-Cox-plot recommended a transformation by square root. The equation of the transformed model is shown in Eq. (2).

2 $$ \begin{aligned} \sqrt {{\mathrm{Ergosterol}}\left[ {{\mathrm{mg}}\:{\mathrm{L}}^{{ - 1}} } \right]} & = + \:4.48 + 0.8480\:{\mathrm{A}} \\ & \quad + 0.5391\:{\mathrm{B}} - 0.0376\:{\mathrm{C}} \\ & \quad - 4.19\:{\mathrm{D}} + 0.5440\:{\mathrm{AB}} \\ & \quad - 2.69\:{\mathrm{AC}} - 1.63\:{\mathrm{AD}} \\ & \quad + 0.6718\:{\mathrm{BC}} + 0.0433\:{\mathrm{CD}} \\ & \quad + 0.0511\:{\mathrm{A}}^{2} - 0.6556\:{\mathrm{B}}^{2} \\ & \quad - 0.7295\:{\mathrm{C}}^{2} - 3.58\:{\mathrm{D}}^{2} \\ & \quad + 1.13\:{\mathrm{A}}^{2} {\mathrm{B}} - 1.59\:{\mathrm{AB}}^{2} \\ & \quad - 2.35\:{\mathrm{AC}}^{2} - 1.16\:{\mathrm{AD}}^{2} \\ & \quad + 2.85\:{\mathrm{C}}^{2} {\mathrm{D}} - 2.04\:{\mathrm{CD}}^{2} \\ \end{aligned} $$

The fit statistic for the model of the ergosterol content per liter is shown in Supplementary Table 11. Like the ergosterol content of the biomass [mg g^− 1^], the predicted R^2^ (0.5) was not as close to the adjusted R^2^ (0.8) as expected. This may again be attributed to a block effect. The signal-noise-ratio was high, which is desirable for navigating the design space.

In the analysis of variance (ANOVA), all terms relevant for the calculation of the model are listed (Supplementary Table 12). Nonsignificant terms were used for hierarchical reasons. The model was significant and showed a non-significant lack of fit.

**Fig. 3:**
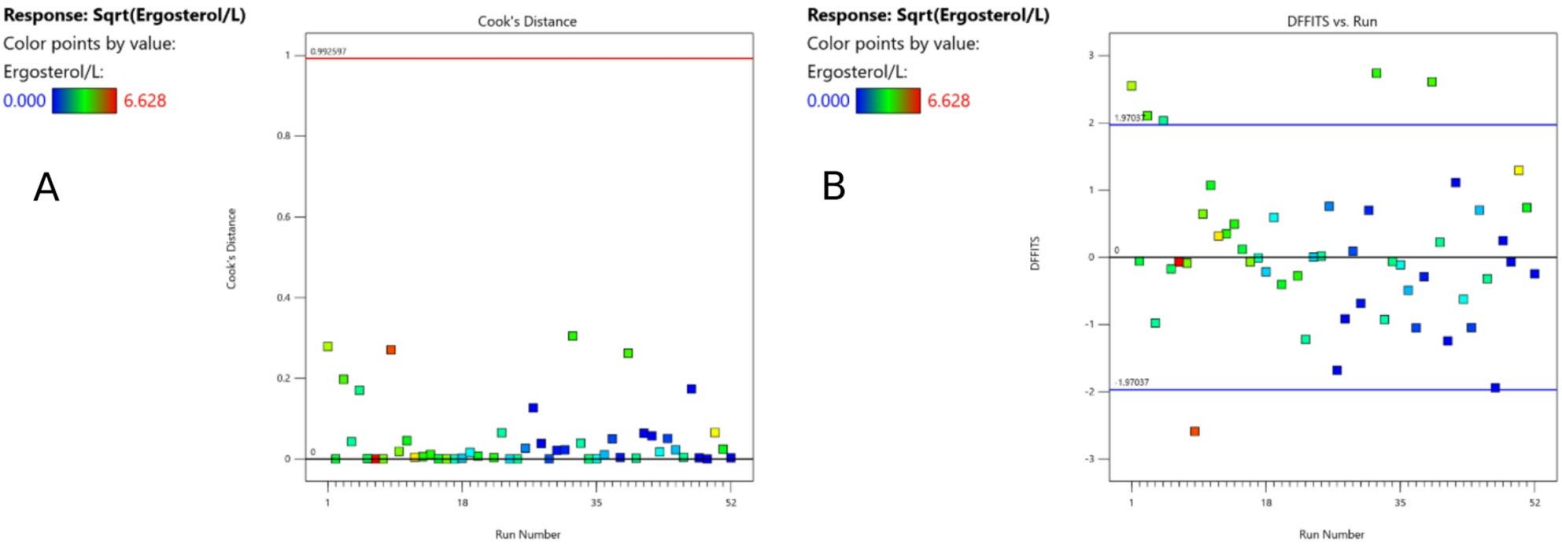
Cook’s distance (**A**) and difference of fits (DFFITS,** B**) for the ergosterol content per liter [mg L^-1^]

**Fig. 4:**
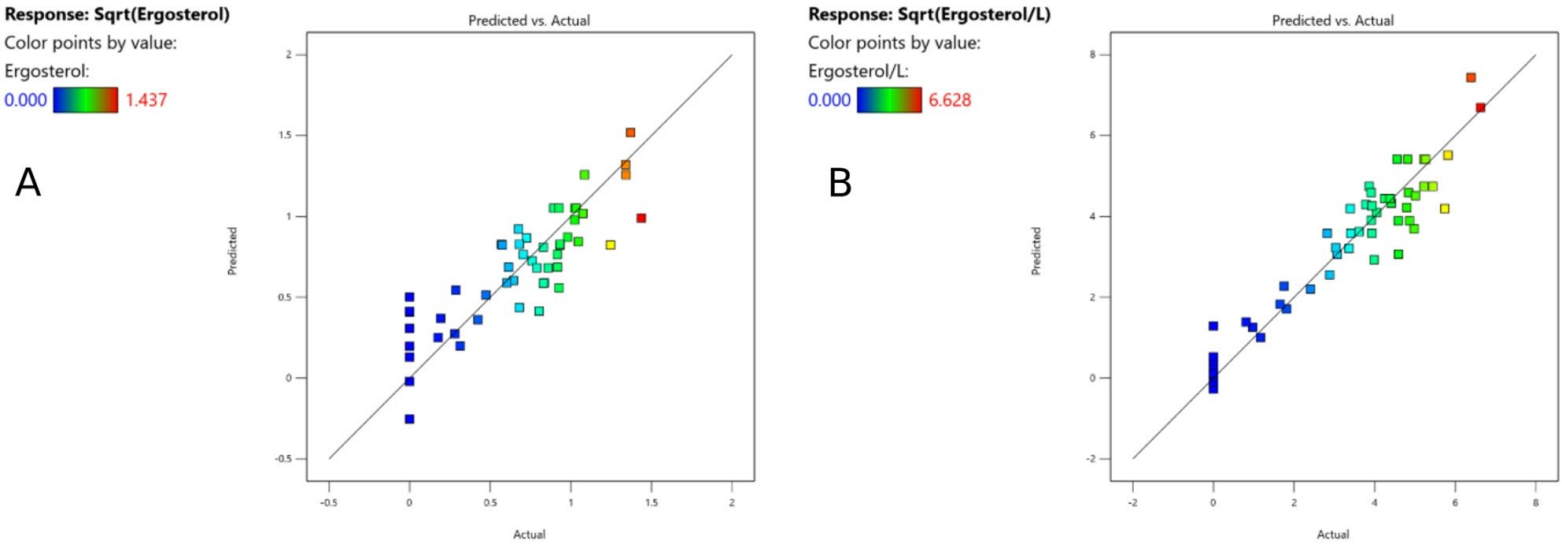
Comparison of the predicted versus actual values for the ergosterol content of the biomass [mg g^-1^] (**A**) and for the ergosterol content per liter [mg L^-1^] (**B**)

In the difference of fits test (DIFFITS) for the ergosterol content per liter [mg L^− 1^], six datapoints were outside the borders, but within the limit in the Cook’s Distance (Fig. 3). In the comparison of predicted versus actual values, no outliers were identified (Fig. 4B). Therefore, all datapoints (except for run 21) were included in the calculation of the model.

**Fig. 5:**
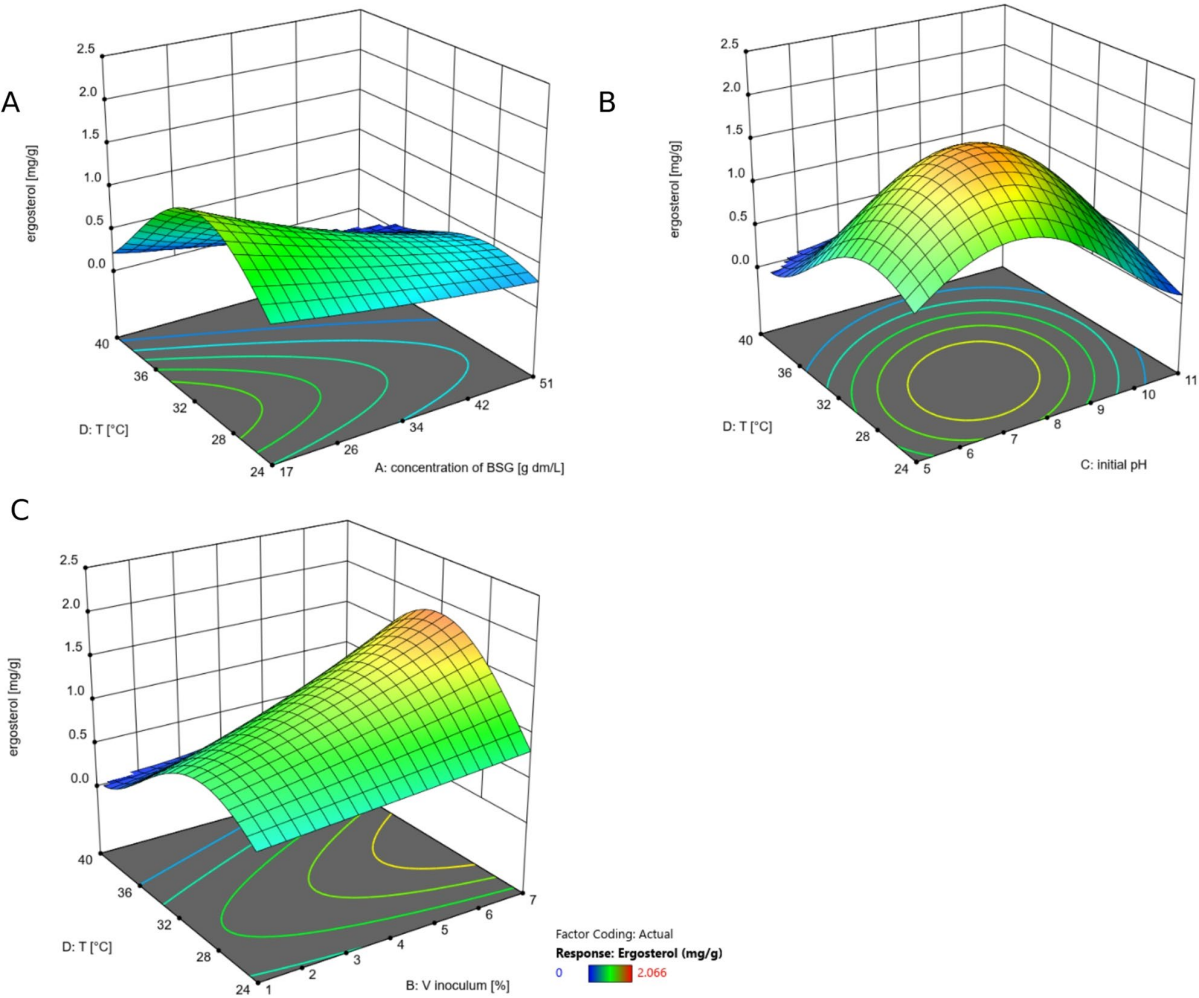
3D plots of the model for the ergosterol content in the biomass [mg g^-1^] (quadratic model, transformed by square root). As only two changing parameters can be shown in one graph, the other parameters were fixed at the final, optimized values. **A**: changes in ergosterol content depending on varying temperature (T) and varying BSG concentration at an initial pH of 9.3 and an inoculum volume of 4.4% (v/v); **B**: changes in ergosterol content depending on varying temperature (T) and varying initial pH at a BSG concentration of 17 g L^-1^ dm and an inoculum volume of 4.4% (v/v); **C**: changes in ergosterol content depending on varying temperature (T) and varying inoculum volume (V Inoculum) at a BSG concentration of 17 g L^-1^ dm and an initial pH of 9.3

All 3D plots of the model for the ergosterol content in the biomass [mg g^− 1^] indicated 30 °C as the optimal cultivation temperature (Fig. 5). The lowest BSG concentration was the best choice for increasing the ergosterol content (Fig. 5A). The plot of the temperature versus the initial pH suggested that a maximum ergosterol content is achieved at 30 °C and a pH of 8 (Fig. 5B). A higher ergosterol content would be possible by further increasing the inoculum volume and keeping the temperature at 30 °C (Fig. 5C). The aim of the optimization was to reduce the inoculum volume to a minimum and therefore, a volume of 4.4% (v/v) was selected.

**Fig. 6:**
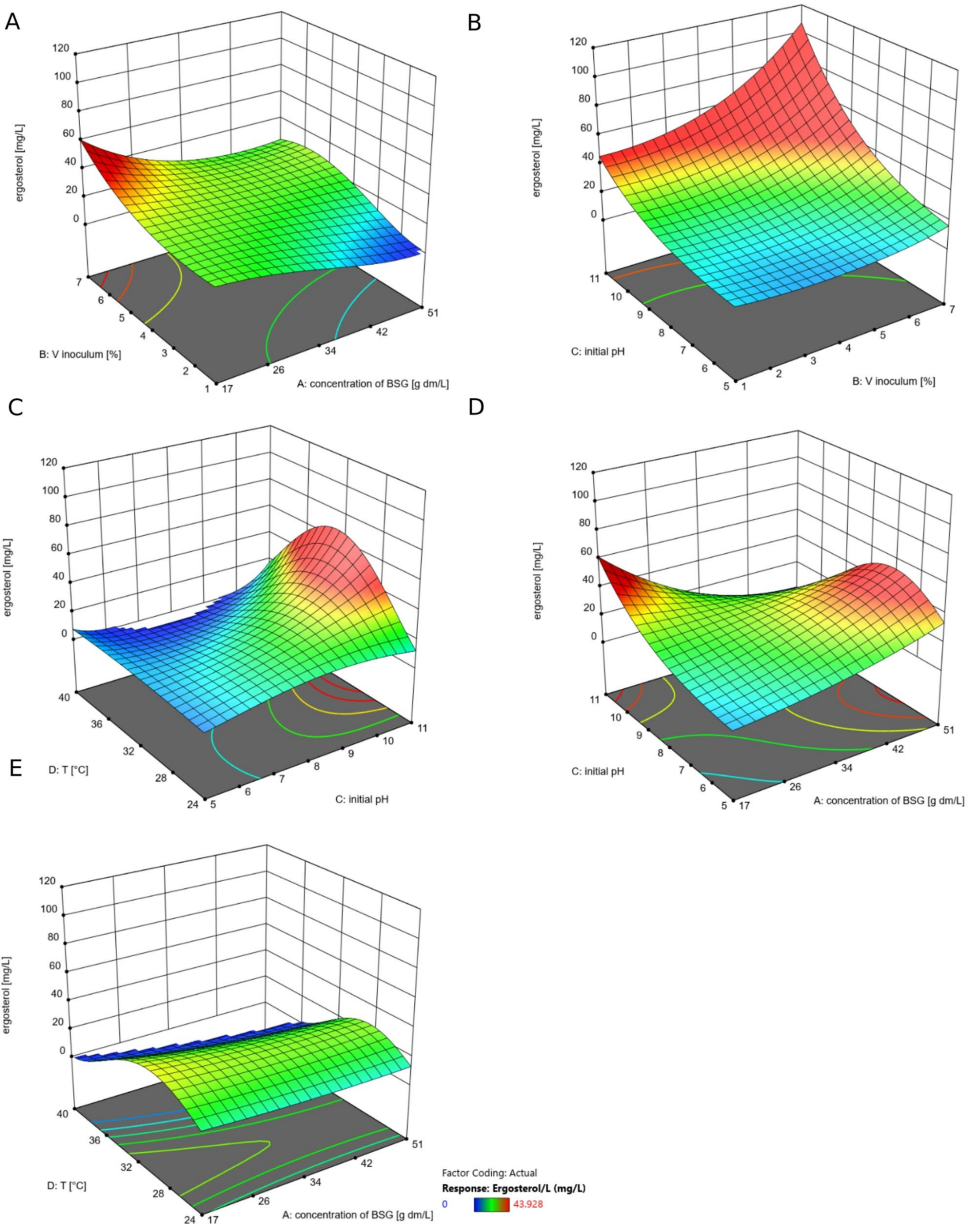
3D plots of the model for the ergosterol content per liter [mg L^-1^] (cubic model, transformed by square root). As only two changing parameters can be shown in one graph, the other parameters were fixed at the final, optimized values. **A**: changes in ergosterol content depending on varying inoculum volume (V Inoculum) and varying BSG concentration at an initial pH of 9.3 and a temperature of 30 °C; **B**: changes in ergosterol content depending on varying inoculum volume (V Inoculum) and varying initial pH at a BSG concentration of 17 g L^-1^ dm and a temperature of 30 °C; **C**: changes in ergosterol content depending on varying initial pH and varying temperature (T) at a BSG concentration of 17 g L^-1^ dm and an inoculum volume of 4.4% (v/v); **D**: changes in ergosterol content depending on varying initial pH and varying BSG concentration at a temperature of 30 °C and an inoculum volume of 4.4% (v/v); **E**: changes in ergosterol content depending on varying temperature (T) and varying BSG concentration at an initial pH of 9.3 and an inoculum volume of 4.4% (v/v)

The 3D plots for the ergosterol content per liter [mg L^− 1^] also showed increasing values with increasing inoculum volume and an optimal temperature of 30 °C (Fig. 6). The interaction between inoculum volume and BSG concentration showed an increasing ergosterol content with increasing inoculum volume and decreasing substrate concentration (Fig. 6A). The same effect was observed in the interaction of BSG concentration and the temperature (Fig. 6E). High ergosterol levels were predicted at high initial pH values in interaction with the inoculum volume (Fig. 6B) or temperatures around 30 °C (Fig. 6C). The interaction between the initial pH value and the BSG concentration revealed high ergosterol contents at high initial pH values and low BSG concentration or low initial pH values and high BSG concentration (Fig. 6D). At a pH of 11, no growth was observed and therefore the experimental plan was slightly modified. Therefore, the model has a high standard deviation at the upper edge of the factor space and this might cause a wrong prediction in this area.

The optimum parameters for maximizing the ergosterol content of the biomass [mg g^− 1^] and the ergosterol content per liter [mg L^− 1^] and simultaneously decreasing the inoculum volume were 17 g L^− 1^ dm BSG concentration, 4.4% (v/v) inoculum volume, an initial pH of 9.3, and a temperature of 30 °C.

A comparison of the values predicted by “Design Expert 13” and the experimentally determined values for the ergosterol contents of the biomass [mg g^− 1^] and per liter [mg L^− 1^] for the optimized fermentation of BSG with *P. ostreatus* confirmed the model. For the ergosterol content [mg g^− 1^] a value of (1.2 ± 0.1) was determined while the predicted value was (1.4 ± 0.6). The experimental value for the ergosterol content [mg L^− 1^] was (16 ± 0) and the prediction was (33 ± 9).

In preliminary experiments, an inoculum volume of 5% (v/v) was used. This was the same volume that was used in other fermentations with *P. ostreatus* in bioreactors (Papaspyridi et al. 2010; Bakratsas et al. 2023, 2024). Considering an upscaling of the fermentation and a possible industrial application, decreasing the inoculum volume saves costs. An even smaller inoculum volume than 4.4% would lead to decreased ergosterol contents. Currently, the fermentation is run in a batch mode. By considering a semi-continuous procedure like repeated-batch fermentation, there would be no need for an inoculum, as an aliquot of the fermentation broth would be replaced by fresh medium. For *Ganoderma lucidum*, *Funalia trogii*, and *Trametes versicolor*, this approach has already been tested to produce exopolysaccharides and laccase. It led to a decrease in fermentation time without negative effects on the fermentation (Birhanli and Yesilada 2010; Wan Mohtar et al. 2016).

In this study, a temperature of 30 °C was considered as optimum. The genus *Pleurotus* is known for its growth in a broad temperature spectrum (Rajarathnam et al. 1987). In previous submerged cultivations of *P. ostreatus*, temperatures between 25 and 28 °C were used, whereas 30 °C has been selected for solid state fermentations (Furlan et al. 1997; Papaspyridi et al. 2010; Hamza et al. 2024).

It was unexpected that the initial pH did not have a significant influence on the fungal content of the biomass. In other studies which improved the culture conditions of *P. ostreatus* by response surface methodology, the influence of the pH was significant (Hamza et al. 2024; Guan et al. 2025). In these studies, an initial pH of 6.5 and 6 was found to be ideal. The focus of Guan’s work was set on the production of biomass and its enrichment with selenium. Hamza et al. focused on the production of biomass, but also on production of exopolysaccharides. They observed no significant influence of the temperature on the model. This contrasts with the results above. Another difference was the medium. Hamza et al. 2024 used a synthetic medium, whereas the medium in this work consisted of non-soluble BSG. Therefore, the medium contained particles and the composition of nutrients in the medium was different. This might explain the insignificance of the initial pH in this study. In most other liquid cultivations of *P. ostreatus*, an optimum initial pH of 6 was reported (Papaspyridi et al. 2010; Bakratsas et al. 2024; Hamza et al. 2024), but an optimal pH of 8 was found for the cultivation of *P. ostreatus* in Nigeria (Jonathan et al. 2011). The optima for initial pH as well as temperature obviously differ with different strains of *P. ostreatus* and the respective culture medium.

The basic pH of 9.3 might affect the composition of the medium as well. Alkaline pretreatments are already known to break down lignin and hemicellulose in hardwood, herbaceous crops and agricultural residues to achieve a higher biomass digestibility (Singh et al. 2015). Even carried out under ambient conditions, lignin was significantly reduced in corn stover by 9% – 46% in 24 h, depending on the NaOH concentration (1.0–7.5% (w/w), Zhu et al. 2010). Chaudhary et al. 2012 observed significant lignin degradation in lignocellulosic biomass at 30 °C within 12–48 h. The higher the NaOH concentration, the more lignin was degraded. They also found that sodium hydroxide pretreatment largely affected hemicellulose solubilization. More than 50% xylan was solubilized after a pretreatment in 7% NaOH for 48 h (Chaudary et al. 2012). Wilkinson et al. found lower lignin and higher glucose contents after alkaline pretreatment of BSG. They found that a treatment with 5% NaOH and a temperature of 50 °C led to a decrease in the lignin content of 30% and to more than 90% theoretical glucose yield. Even 1% NaOH or a short reaction time of 2 h effected changes in the composition (Wilkinson et al. 2014). Adjustment of the pH to 9.3 could have led to a partial degradation of the BSG. That might result in a higher sugar content which may positively influence the fermentation.

In this study, a medium containing solid BSG was used. After mixing with water, there are still particles left in the medium. After the fermentation, the particles cannot be separated from the fungal mycelium, and therefore, the quantification of fungus in the biomass is difficult. Therefore, ergosterol was used as a biomarker to monitor fungal growth. Several researchers have already described the ergosterol content as suitable for determining the fungal content (Manter et al. 2001; Steudler and Bley 2015; Mansoldo et al. 2020). The ergosterol content depends on the medium composition, the species, the oxygen availability, and the temperature (Dexter and Cooke 1984; Manter et al.^ 2001^; Klamer and Bååth 2004; Steudler and Bley 2015). As the concentration of the spent grains varied during optimization, the ergosterol content in relation to the fermentation volume [mg L^− 1^] was also considered in addition to the ergosterol content of the biomass [mg g^− 1^]. The influence of changes in the culture temperature on the ergosterol content was not considered in this study. The fungal content of the fermented biomass was determined using the ergosterol content. For this purpose, the ergosterol content of the pure fungal mycelium, which was cultivated in ME medium, and the ergosterol content of the fermented spent grains were determined. The ergosterol content of the pure mycelium of (6.5 ± 0.2) mg g^− 1^ and of the fermented spent grains of (1.4 ± 0.5) mg g^− 1^ led to a calculated fungal content of about 21% in the fermented spent grains.

### Nutritional analysis of *P. ostreatus* mycelium, BSG and fermented BSG

**Fig. 7:**
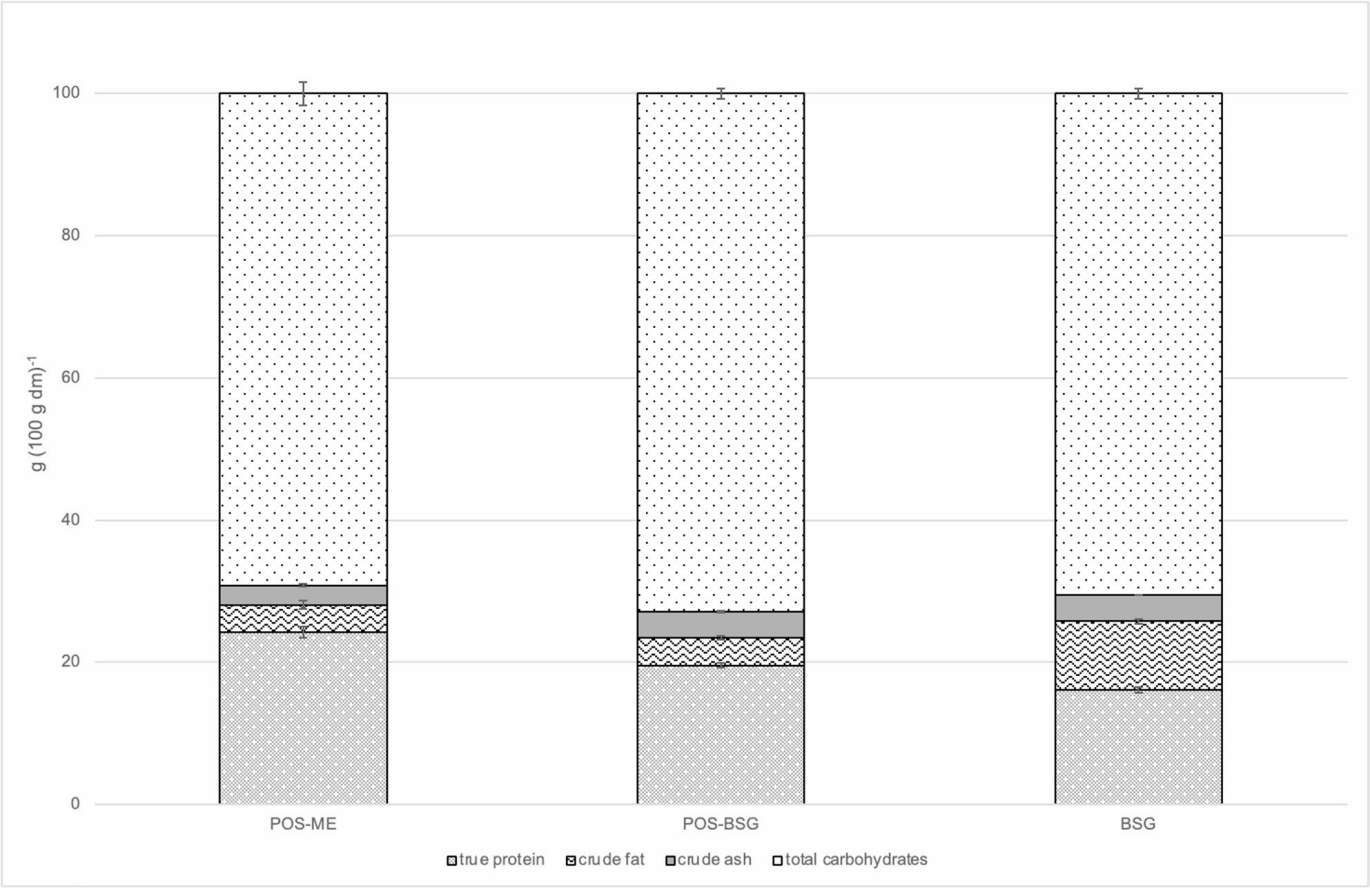
Protein, fat, ash and carbohydrate contents of *Pleurotus ostreatus*mycelium (POS-ME), fermented spent grains (POS-BSG) and non-fermented spent grains (BSG)

After fermentation, the true protein content was increased from 16 g (100 g)^−1^ to 20 g (100 g)^−1^. The crude fat noticeable decreased from 9.6 g (100 g)^−1^ to 3.9 g (100 g)^−1^. This corresponded to the crude fat content of the pure mycelium of *P. ostreatus* (Fig. 7). The ash content did not change as a result of the fermentation. The increased protein content and reduced fat content caused by the fermentation is beneficial from a nutritional point of view.

The analyzed nutritional values of the pure mycelium of *P. ostreatus* largely correspond to published data of Manu-Tawiah and Martin. The protein and fat contents were comparable, while the ash content in their work was higher (Manu-Tawiah and Martin 1987). As the ash content of fungi varies with the salt concentration of the medium, deviations are explainable (Jennison et al. 1957). In general, the nutritional values depend on the strain used, the composition of the media and the cultivation conditions.

The composition of the malt varies depending on the type of beer. The brewing process and the technology used in production also have an influence on the composition of the spent grains (Santos et al. 2003; Robertson et al. 2010). It is therefore difficult to compare the values analyzed here with previously published values. By comparing various literature values, clear differences can be seen. However, the magnitudes of the values from publications match the values analyzed here (Kanauchi et al. 2001; Santos et al. 2003; Mussatto and Roberto 2005; Resconi et al. 2024).

**Fig. 8:**
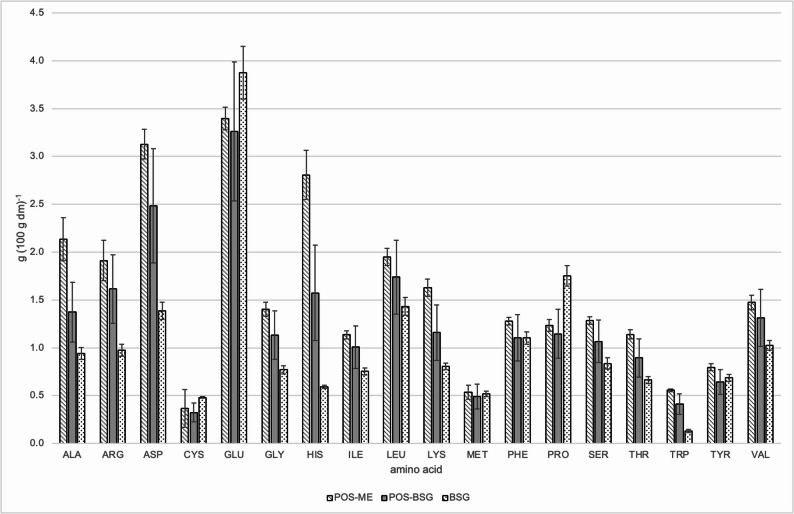
Amino acid profile of *Pleurotus ostreatus* mycelium (POS-ME), fermented spent grains (POS-SBT) and non-fermented spent grains (SBT)

The comparison of the amino acid profiles proved that the concentration of amino acids and therefore the protein content were increased after the fermentation (Fig. 8). It is particularly noticeable that the fermentation significantly increased the tryptophane content. In the unfermented BSG, tryptophane was limiting, while it was no longer after the fermentation. This resulted in an improved biological value that was almost as high as the biological value of the pure mycelium (Table 1). Fungal biomass was built through fermentation. The fungal protein was not limited in tryptophane. The protein of the fermented BSG was composed of fungal protein and remaining spent grain protein in which tryptophane was no longer limiting. In terms of human nutrition and the usage as a food ingredient, the increased true protein content as well as the increased biological value of the protein are highly positive.

**Table 1:** Essential amino acid indices (EAAI), biological values (BV), first and second limiting amino acids (AA) with corresponding chemical scores (CS)

POS-ME	EAAI	BV	1. limiting AA (CS)	2. limiting AA (CS)
	98 ± 3	95 ± 3	Met/Cys (85.3)	Leu (98.7)
POS-BSG	97 ± 2	94 ± 3	Met/Cys (85.5)	Lys (92.4)
BSG	91 ± 2	88 ± 2	Trp (69.5)	Lys (78.3)

POS-ME, pure mycelium of *Pleurotus ostreatus*; POS-BSG, fermented spent grains; BSG, unfermented spent grains

As described for the protein content, the so far published amino acid profiles of the mycelium of *P. ostreatus* differ. In a study reported by Hagar and Cohen-Arazi, aspartic acid was the amino acid with the largest share. Bakratsas et al., however, described glycine as the dominating amino acid. In their work, cysteine and methionine as well as lysine were reported to be limiting (Hadar and Cohen-Arazi 1986; Bakratsas et al. 2023). In this study, glutamic acid, aspartic acid, and histidine were the most abundant amino acids, while cysteine, methionine, and leucine, or lysine were limiting in the pure mycelium or in the spent grains fermented with *P. ostreatus*, respectively. The amino acid profile of brewer’s spent grains was comparable to literature values of Kissel and Prentice, Prentice and Refsguard and Robertson et al. (Prentice and Refsguard 1978; Kissell and Prentice 1979; Robertson et al. 2010). Glutamic acid was the dominant amino acid, as the storage proteins of barley are rich in glutamine (Robertson et al. 2010).

**Fig. 9:**
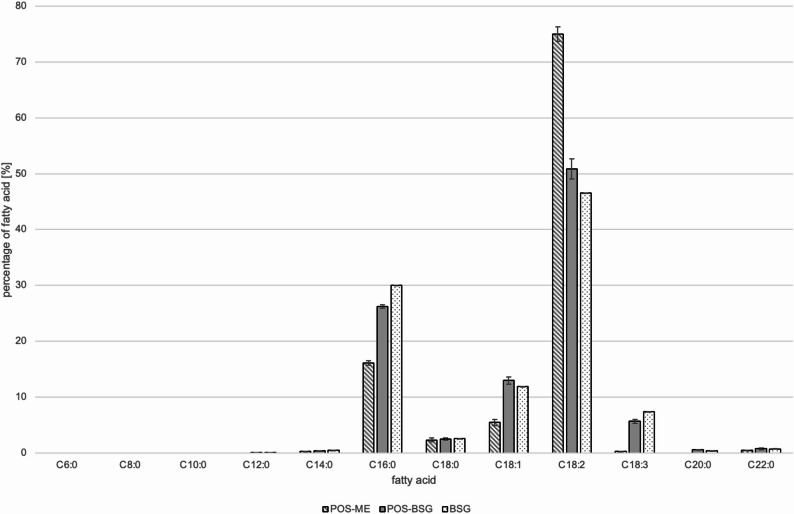
Fatty acid distribution of the pure mycelium of *Pleurotus ostreatus* (POS-ME), fermented brewer’s spent grains (POS-BSG) and non-fermented spent grains (BSG)

The fatty acid profile of the fermented BSG reflected the profiles of the unfermented BSG and the pure mycelium (Fig. 9). Exceptions were oleic acid and arachidic acid, as their shares increased as a result of the fermentation. Due to the fermentation, the amount of unsaturated fatty acids was slightly increased from 67 to 70%. The main fatty acid of all three samples was linoleic acid.

For BSG, the analyzed fatty acid profile well reflected those published by Farcas et al. and Niemi et al. who identified linoleic acid, palmitic acid and oleic acid as the main fatty acids (Niemi et al. 2012; Farcas et al. 2015). The fatty acid profile of the *P. ostreatus* mycelium was comparable to the findings of Dimou et al. who compared different strains of *P. ostreatus* (Dimou et al. 2002). Depending on the strain, the fatty acid composition was different. However, linolic acid was the most abundant fatty acid for all strains.

## Conclusions

In this study, the fermentation of BSG with *P. ostreatus* was established and optimized regarding BSG concentration, inoculum volume, initial pH and temperature. The optimization was performed by using response surface methodology. The inoculum volume could be decreased in comparison to average values of the literature and the optimized fermentation conditions led to a fungal content of 21% after a cultivation time of 4 days. The fermentation increased the protein content and the biological value. The fermented spent grains could be used as an ingredient for meat alternatives like vegan sausages, chunks or minced meat. As the increased protein quality and quantity is a result of the formed fungal biomass, a further improvement of the fermentation and a higher fungal content might further improve the protein quality and quantity. The fermentation product still contains the husks of the spent grains. This may negatively affect the mouth feeling as the consistency was described as woody and fibrous. The fermented BSG was brown in color and had a mushroom-like and slightly earthy and savory odor. Since it is to be used as an ingredient in meat alternatives and probably will be cooked, the color is suitable. Meat alternatives are typically seasoned, so the faint odor of the fermentation product will probably no longer be noticeable in the processed product. Overall, the fermentation upcycled the BSG in terms of protein quality and quantity, which is desirable for use as food. Further research could further improve the fermentation product, especially regarding the mouth feeling.

## Supplementary Information

Below is the link to the electronic supplementary material.

Supplementary Material 1.

## Data Availability

All data generated or analysed during this study are included in this published article and its supplementary information file.
