# Supplementary material for: Fermentation of brewer’s spent grains by Pleurotus ostreatus: process optimization by response surface methodology
Source: Bioresour Bioprocess. 2026 May 14;13(1):72. doi: 10.1186/s40643-026-01018-3 (PMC13176399; doi:10.1186/s40643-026-01018-3)
Supplement: Supplementary file 1 — Supplementary Material 1. [file 40643_2026_1018_MOESM1_ESM.docx]

# Fermentation of brewer’s spent grains by *Pleurotus ostreatus* – process optimization by response surface methodology

Victoria-Luisa Hrazdil^a^, Paula Hallmann^a^, Josephine Dresler^b^, Marco A. Fraatz^a^, Holger Zorn^a, b^*

^a^Institute of Food Chemistry and Food Biotechnology, Justus Liebig University Giessen, Heinrich-Buff-Ring 17, 35392 Giessen, Germany

^b^[Fraunhofer Institute for Molecular Biology and Applied Ecology,](https://www.ime.fraunhofer.de/en.html) Ohlebergsweg 12, 35392 Giessen, Germany

ORCID

Holger Zorn: 0000-0002-8383-8196

* Corresponding author. Tel.: +49 (0) 641 99 34900

E-mail address: holger.zorn@uni-giessen.de

## Materials and Methods

### Culture medium and bioreactor

Supplementary table1: Bioreactors used for the fermentations

| SciVario® twin (Eppendorf SE, Jülich (DE)) | |
| --- | --- |
| culture vessel | DS1500 (working volume 0.4 – 1.5 L, cell culture) |
| pH-electrode | EasyFerm Plus (325 mm, Hamilton Bonaduz, Bonaduz (CH)) |
| DO-sensor (optical) | VisiFerm RS485 (325 mm, H2 cap, Hamilton Bonaduz AG, Bonaduz (CH)) |
| gassing type | submerged |
| sparger | L-sparger |
| stirrer | 2 pitch blade impeller, 1 Rushton turbine |
| software for recording | DASware® control (version 6.5.0.0) |

Supplementary table 2: Cascade used for the fermentations

|  | Controller output [%] | Setpoint |
| --- | --- | --- |
| Agitation | 0 | 200 rpm |
|  | 50 | 500 rpm |
| Oxygen concentration | 0 | 21% |
|  | 100 | 21% |
| Flow | 0 | 5.5 L h^-1^ |
|  | 100 | 15 L h^-1^ |

### Experimental design

Supplementary table 3: Real and coded factors and their ranges

| Factor | Name | Unit | Type | Sub Type | Minimum | Maximum | Coded Low | Coded High |
| --- | --- | --- | --- | --- | --- | --- | --- | --- |
| A | Conc. of BSG | g L^‑1^ dm | Numeric | Continuous | 17.0 | 51.0 | -1 ↔ 17.0 | +1 ↔ 51.0 |
| B | V inoculum | % | Numeric | Discrete | 1.0 | 7.0 | -1 ↔ 1.0 | +1 ↔ 7.0 |
| C | Initial pH |  | Numeric | Continuous | 4.2 | 11.0 | -1 ↔ 5.0 | +1 ↔ 11.0 |
| D | T | °C | Numeric | Continuous | 20.0 | 40.0 | -1 ↔ 24.0 | +1 ↔ 40.0 |

Supplementary table 4: Experimental values of ergosterol for the different runs with modified parameters

| Block | Run | Concentration of BSG [g L^‑1^ dm] | Inoculum volume [% (v/v)] | Initial pH | | T [°C] | Ergosterol [mg g^‑1^] | Ergosterol [mg L^‑1^] |
| --- | --- | --- | --- | --- | --- | --- | --- | --- |
| Block 1 | 1 | 51 | 7.0 | 4.5 | 30.0 | | 0.69 | 29.63 |
| Block 1 | 2 | 34 | 1.0 | 5.2 | 30.0 | | 0.74 | 19.19 |
| Block 1 | 3 | 17 | 1.0 | 7.0 | 27.2 | | 1.80 | 25.13 |
| Block 1 | 4 | 17 | 7.0 | 5.3 | 25.1 | | 1.18 | 15.31 |
| Block 1 | 5 | 41 | 1.0 | 7.0 | 20.0 | | 0.50 | 15.90 |
| Block 1 | 6 | 34 | 1.0 | 5.2 | 30.0 | | 0.62 | 18.07 |
| Block 1 | 7 | 44 | 5.5 | 6.1 | 30.0 | | 1.16 | 43.93 |
| Block 1 | 8 | 34 | 4.0 | 5.6 | 25.0 | | 1.05 | 27.27 |
| Block 1 | 9 | 51 | 7.0 | 7.0 | 22.9 | | 1.05 | 40.83 |
| Block 1 | 10 | 51 | 4.0 | 5.2 | 20.0 | | 0.70 | 27.26 |
| Block 1 | 11 | 34 | 4.0 | 4.2 | 25.0 | | 0.84 | 21.08 |
| Block 1 | 12 | 27 | 7.0 | 7.0 | 30.0 | | 1.88 | 33.84 |
| Block 1 | 13 | 17 | 7.0 | 5.3 | 25.1 | | 1.80 | 23.41 |
| Block 1 | 14 | 33 | 7.0 | 6.0 | 21.0 | | 0.96 | 23.02 |
| Block 1 | 15 | 48 | 1.0 | 6.0 | 25.2 | | 0.53 | 19.46 |
| Block 1 | 16 | 34 | 4.0 | 5.6 | 25.0 | | 1.07 | 27.72 |
| Block 1 | 17 | 25 | 2.5 | 6.1 | 20.0 | | 0.69 | 13.07 |
| Block 1 | 18 | 21 | 1.0 | 4.2 | 26.2 | | 0.58 | 9.22 |
| Block 1 | 19 | 17 | 4.0 | 4.2 | 30.0 | | 0.87 | 11.31 |
| Block 1 | 20 | 34 | 4.0 | 5.6 | 25.0 | | 0.80 | 20.75 |
| ~~Block 1~~ | ~~21~~ | ~~51~~ | ~~2.5~~ | ~~7.0~~ | ~~30.0~~ | | ~~0.00~~ | ~~0.00~~ |
| Block 1 | 22 | 34 | 4.0 | 5.6 | 25.0 | | 0.86 | 23.19 |
| Block 1 | 23 | 51 | 4.0 | 5.2 | 20.0 | | 0.36 | 14.92 |
| Block 1 | 24 | 34 | 4.0 | 4.2 | 25.0 | | 0.38 | 9.43 |
| Block 1 | 25 | 17 | 5.5 | 7.0 | 20.0 | | 1.10 | 15.33 |
| Block 1 | 26 | 17 | 1.0 | 4.5 | 20.0 | | 0.41 | 5.80 |
| Block 1 | 27 | 51 | 1.0 | 4.2 | 25.0 | | 0.00 | 0.00 |
| Block 1 | 28 | 34 | 7.0 | 4.2 | 20.0 | | 0.00 | 0.00 |
| Block 2 | 29 | 25 | 2.5 | 6.3 | 35.0 | | 0.18 | 3.29 |
| Block 2 | 30 | 28 | 1.0 | 9.0 | 35.0 | | 0.03 | 0.66 |
| Block 2 | 31 | 51 | 7.0 | 9.0 | 35.0 | | 0.04 | 1.37 |
| Block 2 | 32 | 17 | 5.5 | 9.0 | 35.0 | | 2.07 | 23.68 |
| Block 2 | 33 | 17 | 5.5 | 9.0 | 24.0 | | 0.84 | 14.29 |
| Block 2 | 34 | 34 | 7.0 | 6.0 | 26.8 | | 0.46 | 16.43 |
| Block 2 | 35 | 26 | 2.5 | 6.0 | 26.8 | | 0.46 | 11.60 |
| Block 2 | 36 | 26 | 2.5 | 6.0 | 26.8 | | 0.32 | 7.97 |
| Block 2 | 37 | 51 | 2.5 | 9.0 | 31.3 | | 0.08 | 3.07 |
| Block 2 | 38 | 17 | 2.5 | 6.3 | 35.0 | | 0.08 | 0.95 |
| Block 2 | 39 | 51 | 4.0 | 9.0 | 24.0 | | 0.65 | 24.81 |
| Block 2 | 40 | 26 | 2.5 | 6.0 | 26.8 | | 0.87 | 15.45 |
| Block 3 | 41 | 40 | 4.0 | 10.0 | 24.0 | | 0.00 | 0.00 |
| Block 3 | 42 | 28 | 2.5 | 11.0 | 24.0 | | 0.00 | 0.00 |
| Block 3 | 43 | 34 | 4.0 | 8.0 | 32.0 | | 0.33 | 11.53 |
| Block 3 | 44 | 28 | 7.0 | 5.0 | 37.0 | | 0.10 | 2.73 |
| Block 3 | 45 | 40 | 1.0 | 9.0 | 30.0 | | 0.22 | 8.32 |
| Block 3 | 46 | 40 | 4.0 | 5.0 | 30.0 | | 0.46 | 15.48 |
| Block 3 | 47 | 17 | 1.0 | 10.0 | 40.0 | | 0.00 | 0.00 |
| Block 3 | 48 | 40 | 7.0 | 10.5 | 24.0 | | 0.00 | 0.00 |
| Block 3 | 49 | 17 | 4.0 | 7.0 | 38.0 | | 0.00 | 0.00 |
| Block 3 | 50 | 34 | 4.0 | 8.0 | 32.0 | | 1.55 | 32.94 |
| Block 3 | 51 | 40 | 4.0 | 5.0 | 24.0 | | 0.86 | 21.03 |
| Block 3 | 52 | 34 | 4.0 | 10.0 | 37.0 | | 0.00 | 0.00 |

### Determination of crude protein

Supplementary table 5: Parameters of the steam distillation apparatus used for the determination of crude protein

| parameter | value |
| --- | --- |
| addition of water | 90 mL |
| addition of NaOH (32%) | 80 mL |
| reaction time | 0 s |
| destillation time | 240 s |
| steam power | 100% |
| addition of boric acid | 80 mL |
| sample suction | 30 s |

### Determination of crude fat

Supplementary table 6: Parameter of the digestion apparatus for the crude fat determination

| phase | parameter | value |
| --- | --- | --- |
| levels | hydrochloric acid | 125 mL |
|  | water | 125 mL |
| heating phase | heat-up phase | 10 min |
|  | boiling phase I | 26 min |
|  |  | 50% |
|  | boiling phase II | 30 min |
|  |  | 70% |
|  | cooling phase | 20 min |
| filter – humidification | amount of cycles | 3 |
|  | volume per cycle | 30 mL |
| filtration phase | waiting time | 5 s |
|  | rinsing cycles | 18 |
|  | line pipe opening time | 200 ms |
| filtration phase | sample rinsing time | 10 s |
|  | sample rinsing | 50 mL |
|  | cooler rinsing | 40 mL |
|  | filter rinsing | 40 mL |

Supplementary table 7: Parameter for lipid extraction

| parameter | value |
| --- | --- |
| petroleum ether 35/65 | 155 mL |
| T-category | 200 °C |
| extraction temperature | 150 °C |
| lowering interval | 4 min |
| lowering impulse | 2 s |
| boiling phase | 30 min |
| distillation intervals AA | 5 |
| extraction time | 90 min |
| distillation intervals AB | 4 |
| distillation AC | 5 min |

### Determination of amino acid composition

Supplementary table 8: Parameter for amino acid analysis

| parameter | amino acid analyzer S433 (SYKAM GmbH, Eresing, DE) |
| --- | --- |
| precolumn | ammonia filter column LCA K13/Na (4,6 x 100 mm) |
| injection volume | 50 µL |
| seperation column | cation exchange column LCA K13/Na (PEEK, 4,6 x 175 mm) |
| eluents | A: Na-citrate buffer A1 (0,12 N, pH 3,45, SYKAM)  B: Na-citrate buffer B1 (0,20 N, pH 10,85, SYKAM)  C: regeneration solution (0,2 g L^‑1^ EDTA in 0,5 M NaOH)  flow rate: 0,45 mL min^‑1^ |
| amino module | ninhydrin (0,1 M in methanol)  flow rate: 0,25 mL min^‑1^ |
| reactor temperature | 130 °C |

## Results and discussion

### Optimization of the fermentation with DOE

Supplementary table 9: Fit statistic of the model for the ergosterol content [mg g^-1^], transformed by square root

| Coefficient of determination R^2^ | 0.6302 |
| --- | --- |
| Adjusted R_adj_^2^ | 0.5449 |
| Predicted R_pred_^2^ | 0.2458 |
| Signal-to-noise-ratio S/N | 14.9810 |

Supplementary table 10: ANOVA (analysis of variance) for the model of the ergosterol content of the biomass [mg g^‑1^] (quadratic model transformed by square root); A: Concentration of BSG, B: Inoculum volume, C: Initial pH, D: Temperature

|  | F-value | p-value |  |
| --- | --- | --- | --- |
| **Model** | 7.39 | < 0.0001 | significant |
| A-Concentration of BSG | 14.62 | 0.0005 | significant |
| B-Inoculum volume | 11.69 | 0.0015 | significant |
| C-Initial pH | 0.3987 | 0.5315 | not significant |
| D-Temperature | 21.55 | < 0.0001 | significant |
| AD | 3.60 | 0.0651 | not significant |
| BD | 4.67 | 0.0369 | significant |
| CD | 5.54 | 0.0237 | significant |
| C² | 27.09 | < 0.0001 | significant |
| D² | 26.56 | < 0.0001 | significant |
| **Residual** |  |  |  |
| Lack of Fit | 1.57 | 0.2303 | not significant |

Supplementary table 11: Fit statistic for the model of the ergosterol content per liter [mg L^-1^], transformed by square root

| Coefficient of determination R^2^ | 0.8643 |
| --- | --- |
| Adjusted R_adj_^2^ | 0.7754 |
| Predicted R_pred_^2^ | 0.4959 |
| Signal-to-noise-ratio S/N | 14.5590 |

Supplementary table 12: ANOVA (analysis of variance) for the model of the ergosterol content per liter [mg L^-1^] (cubic model, transformed by square root); A: Concentration of BSG, B: Inoculum volume, C: Initial pH, D: Temperature

|  | F-value | p-value |  |
| --- | --- | --- | --- |
| **Model** | 9.72 | < 0.0001 | significant |
| A-Concentration of BSG | 3.07 | 0.0905 | not significant |
| B-Inoculum volume | 3.26 | 0.0815 | not significant |
| C-Initial pH | 0.0021 | 0.9638 | not significant |
| D-Temperature | 25.27 | < 0.0001 | significant |
| AB | 4.62 | 0.0400 | significant |
| AC | 20.60 | < 0.0001 | significant |
| AD | 5.00 | 0.0332 | significant |
| BC | 4.57 | 0.0411 | significant |
| CD | 0.0078 | 0.9300 | not significant |
| A² | 0.0278 | 0.8688 | not significant |
| B² | 5.76 | 0.0230 | significant |
| C² | 0.6909 | 0.4126 | not significant |
| D² | 23.10 | < 0.0001 | significant |
| A²B | 5.91 | 0.0215 | significant |
| AB² | 11.74 | 0.0018 | significant |
| AC² | 13.63 | 0.0009 | significant |
| AD² | 5.13 | 0.0311 | significant |
| C²D | 9.31 | 0.0048 | significant |
| CD² | 5.54 | 0.0255 | significant |
| **Residual** |  |  |  |
| Lack of Fit | 1.07 | 0.4769 | not significant |


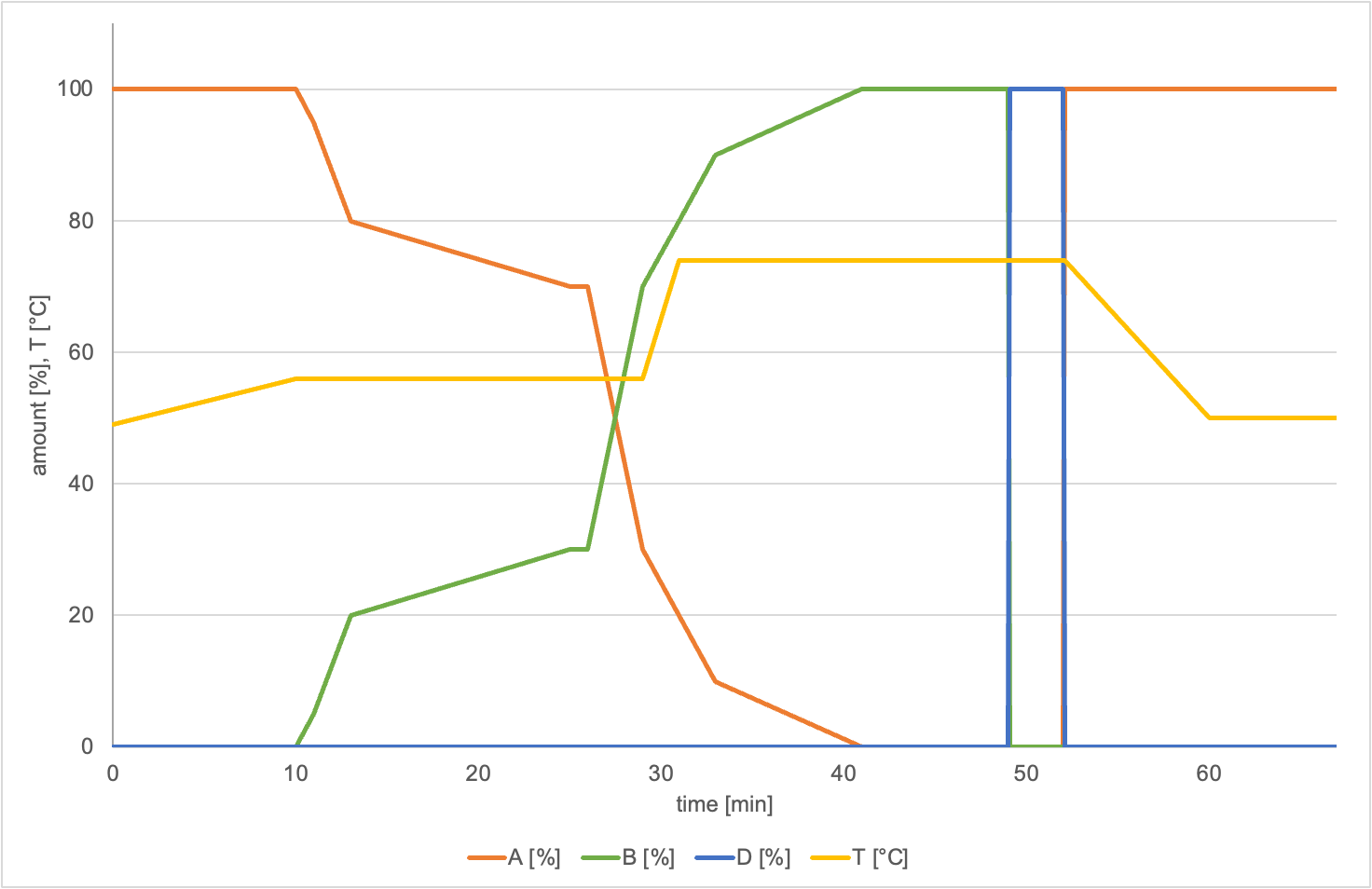

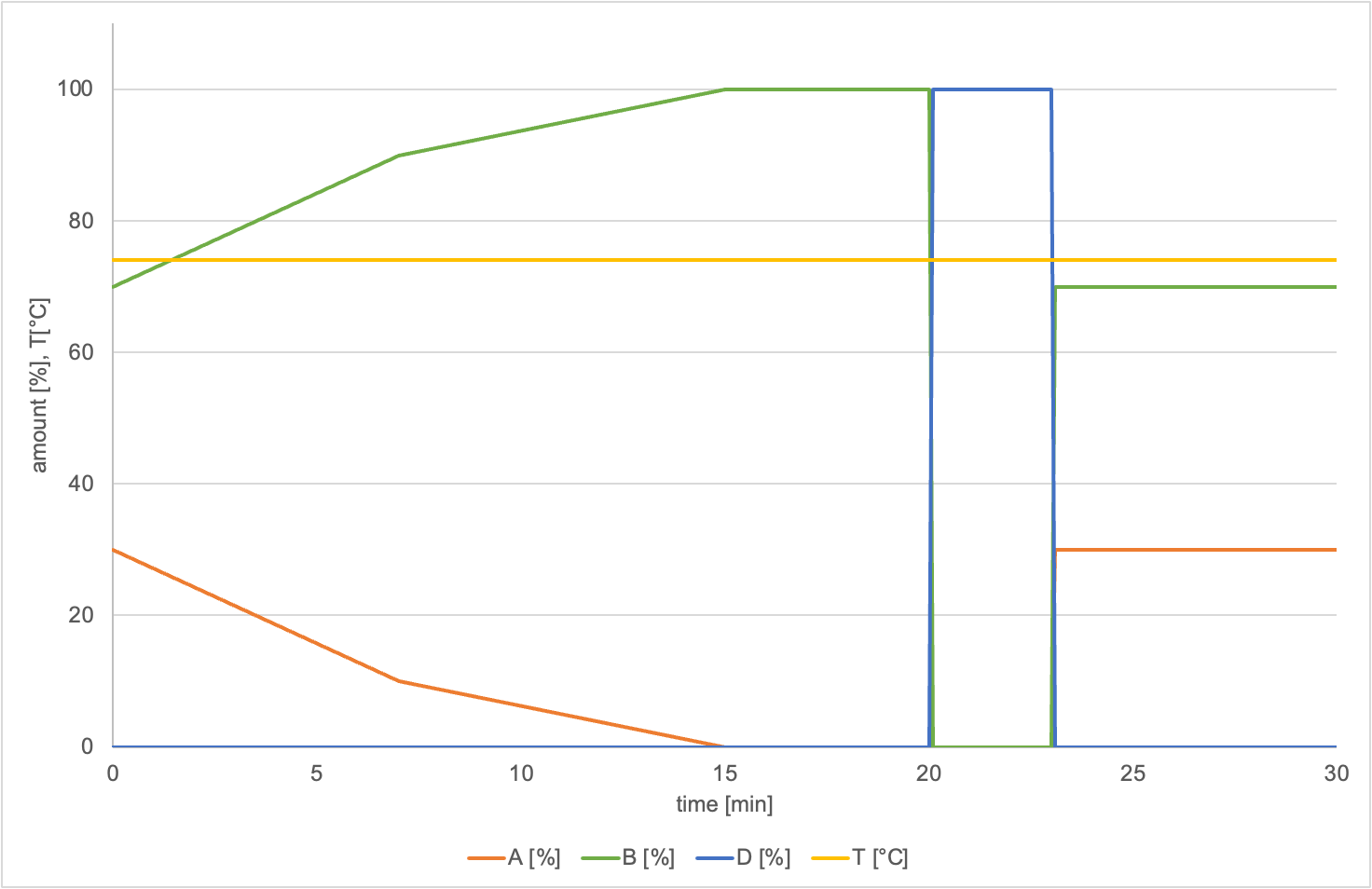


Supplementary figure1: Gradient and oven temperature for the amino acid analysis. Left side: total amino acids, MET and CYS; right side: TRP
